# Identity-by-Descent-Based Phasing and Imputation in Founder Populations Using Graphical Models

**DOI:** 10.1002/gepi.20635

**Published:** 2011-10-17

**Authors:** Kimmo Palin, Harry Campbell, Alan F Wright, James F Wilson, Richard Durbin

**Affiliations:** 1 Wellcome Trust Sanger Institute, Wellcome Trust Genome CampusHinxton, Cambridge, United Kingdom; 2 Centre for Population Health Sciences, University of EdinburghTeviot Place, Edinburgh, United Kingdom; 3 Medical Research Council Human Genetics Unit, Institute of Genetics and Molecular Medicine, Western General HospitalEdinburgh, United Kingdom

**Keywords:** haplotype, population isolate, long-range phasing, Bayesian network

## Abstract

Accurate knowledge of haplotypes, the combination of alleles co-residing on a single copy of a chromosome, enables powerful gene mapping and sequence imputation methods. Since humans are diploid, haplotypes must be derived from genotypes by a phasing process. In this study, we present a new computational model for haplotype phasing based on pairwise sharing of haplotypes inferred to be Identical-By-Descent (IBD). We apply the Bayesian network based model in a new phasing algorithm, called systematic long-range phasing (SLRP), that can capitalize on the close genetic relationships in isolated founder populations, and show with simulated and real genome-wide genotype data that SLRP substantially reduces the rate of phasing errors compared to previous phasing algorithms. Furthermore, the method accurately identifies regions of IBD, enabling linkage-like studies without pedigrees, and can be used to impute most genotypes with very low error rate. *Genet. Epidemiol*. 2011. © 2011 Wiley Periodicals, Inc.35:853-860, 2011

## INTRODUCTION

The haplotype phase, the co-occurrence of alleles on a chromosome, is a vital piece of information underlying many powerful gene mapping methods including haplotype association, linkage, and genotype imputation [de Bakker et al., 2005]. While high-density genotyping arrays provide accurate genotypes on the order of a million single nucleotide polymorphisms (SNPs) from a single individual, they do not reveal which alleles of heterozygous sites reside in the same DNA molecule.

If samples are appropriately related in a known pedigree, phase can be inferred by segregation, but often samples are not closely related. In the absence of efficient experimental methods for haplotype inference [Fan et al., 2010; Kitzman et al., 2010], the past decade has seen the development of several computational approaches for estimating the haplotype phase. These include imperfect phylogeny [Halperin and Eskin, 2004], haplotype copying [Li and Stephens, 2003], and localized clustering methods [Browning and Browning, 2007; Koivisto et al., 2003; Scheet and Stephens, 2006]. All these assume that one is analyzing a relatively small, homogenous sample of individuals from a large outbred population. In such a case, the individuals are assumed to be distantly related, descending from a small set of founders hundreds or thousands of generations ago and possibly having undergone observed mutations since their most recent common ancestor. These assumptions hold to a large extent in many well-studied populations and sample sets, such as 120 “Utah residents with ancestry from northern and western Europe” [Frazer et al., 2007] or 3,000 people “born in England, Wales or Scotland” [The Wellcome Trust Case Control Consortium, 2007]. In contrast, these assumptions are not met when a large enough proportion of the population has been genotyped, as is increasingly the case for isolated founder populations such as Iceland [Kong et al., 2008] or Finland [Jakkula et al., 2008]. As attention shifts back from common to rare variants [Manolio et al., 2009] isolates with founder effects have revived potential for genetic research, because rare variants can more readily drift to higher frequencies hence facilitating phenotype mapping [Holm et al., 2011; Kenny et al., 2009].

Recently, Kong et al. [2008] introduced a “long-range phasing” approach for inferring the haplotype phase in sample sets that cover a relatively large proportion of the population. Their key insight was that, while it is improbable for a distantly related pair of individuals, say third or fourth cousins, to share a genomic locus identical-by-descent (IBD) from this recent common ancestor, if they do share such a locus, they are likely to share quite a long stretch of neighboring loci, 10–12.5 cM on average. This long segment of DNA is likely to contain a thousand or more SNP markers on a commercial genotyping array making it possible to deduce IBD from the identity-by-state (IBS) of the observed markers.

Kong et al. demonstrated the method on genotype data which covered more than 10% of the census population of Iceland where they estimated that on average 19 genotyped individuals share a genomic locus IBD from a historical common ancestor. This large sample from the relatively small population of Iceland allowed them to implement the long-range phasing with a fairly straightforward rule-based method which does not take full use of the data but disregards partly inconsistent information [Hickey et al., 2011; Kong et al., 2008]. Their method phases a fixed segment of a chromosome at a time, making it hard to locate recombination sites and phase markers around them.

Recently, Genovese et al. [2010] presented a Hidden Markov Model (HMM) for IBD detection between a pair of diploid individuals. For phasing multiple individuals, they formulate a combinatorial problem which again is a break from probabilistic framework, with similar issues as with the Kong et al. method.

These observations prompt the development of a fully probabilistic model for accurate haplotype phasing and IBD inference in a large, densely genotyped and relatively closely related set of individuals. Others have recently described methods for IBD inference in such sample sets [Browning and Browning, 2010, 2011; Gusev et al., 2009]. Here, we introduce a new model for haplotype phasing, which is inspired by the long-range phasing approach of Kong et al. [2008] and by Gallager codes [1962] from coding theory.

We demonstrate that our method is able to provide accurate genome-wide haplotype phase and IBD relationship information, both in a simulated founder population and in real data from the Orkney islands. We also contribute an open-source software implementing the described method. Our main contribution is the model-based approach for long-range phasing, which has intuitive parameterization, probabilistic output, and clear structure, which can provide insights to distribution of haplotypes and lends itself to further methods development.

## MATERIAL AND METHODS

### A GRAPHICAL MODEL FOR IBD AND HAPLOTYPE PHASE

Following the long-range phasing approach [Kong et al., 2008], we compare pairs of individuals to locate genomic segments likely to descend from a recent common ancestor without internal recombination events. Starting from this information, we infer the phase of the heterozygous markers and refine IBD constraints between the haplotypes of all pairs of individuals. More technically, we use the HMM to approximate the IBD process along each pair of diploid genomes [Genovese et al., 2010] and combine all pairwise HMMs to form a Bayesian network. The most probable haplotype phases and IBD relationships are inferred from the unobserved variables of the network with the Min-Sum (also known as Max-Product or Viterbi) algorithm [Kschischang et al., 2001].

We approximate the IBD process along a pair of diploid genomes with a five state continuous time Markov model running along the chromosome. The states of the model capture a subset of the possible IBD relationships between the chromosomes; there is one state for the case when the two individuals do not share a haplotype IBD, and one for each of the four cases when the two individuals share exactly one haplotype IBD. While there are 15 different ways for a subset of four haplotypes to be IBD [Thompson, 2008], the others either involve the two haplotypes within an individual being IBD, in which case the phasing is trivial, or contain one of our four cases as a subcase that contains all the phasing information. The five-state model therefore allows substantial computational simplification while preserving sufficient detail for our main goal of long-range haplotype phasing.

The IBD process is parameterized with rate *g*, of moving from the non-IBD state to an IBD-state, and with rate *l*, of moving from an IBD state to the non-IBD state. The rates *g* and *l* also define the expected lengths of IBD and non-IBD segments given IBS. Since the IBD process is continuous time along the chromosome, it is possible for the IBD relationship to “flip” to a different pair of haplotypes between adjacent pairs of markers. The transition probabilities are calculated as exp(*tQ*), where *Q* is the rate matrix and *t* is the genetic distance between adjacent markers estimated from the fine scale genetic map [Myers et al., 2005].

We limit the probability of moving to an IBD state between adjacent markers to the unconditional probability of IBD in the population, i.e. the kinship coefficient. For isolated populations with high kinship and dense genotyping arrays, this limit only affects a small portion of distantly positioned markers.

On each SNP marker, the hidden state of the HMM emits a pair of diplotypes, that is ordered pairs of alleles, one diplotype for each individual. The emission probabilities follow the allele frequencies observed in the sample and Hardy-Weinberg equilibrium, given the IBS constraint imposed by the IBD status of the hidden state. For example, take one individual who is homozygous for the minor allele and another one who is heterozygous. If they are IBD on one chromosome, they must share the minor allele and the emission probability for the pair of diplotypes is *f*^2^(1 − *f*) with minor allele frequency of *f*. If they are not IBD, the probability is *f*^3^(1 − *f*).

**Fig. 1:**
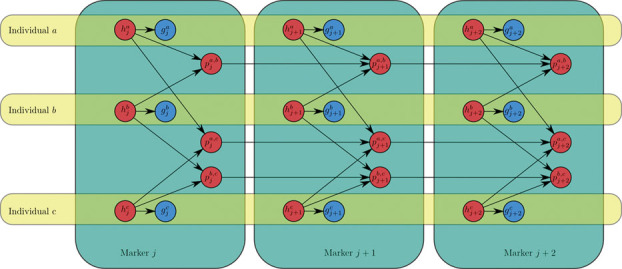
Bayesian network for the SLRP model of haplotype phasing and IBD inference. The observed genotype of an individual *a* at marker *j* is in variable 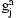, which depends on the diplotype 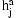. Variable 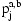 indicates the type of IBD between a pair of individuals *a* and *b* at the marker *j*. IBD, identity-by-descent; SLRP, systematic long-range phasing.

To use the model for phasing, we combine the HMMs for all pairs of individuals into a Bayesian network. The network illustrated in Figure 1, includes observed variables *g* for the genotypes and hidden variables *h* for the diplotypes and *p* for the IBD relationship between pairs of individuals. A variable 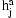 encodes the diplotype for individual *a* on marker *j*. The distribution of the observed genotype 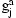 depends essentially deterministically on the underlying diplotype 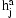 but allowing for some noise from the genotyping assay. The network also includes an IBD variable 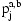 for each SNP *j* and pair of individuals *a* and *b*. This variable encodes the IBD relationship between the two individuals at marker *j*.

The conditional probability of 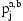 given the diplotypes on current marker and the IBD state on the previous marker is calculated by inverting the emission probabilities of the HMM described above via a version of Bayes rule that also incorporates the transition probabilities


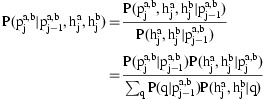


To complete the model, the diplotypes 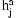 are given an uniform prior. A more sophisticated prior for the diplotypes might help with imputation of missing data, but choice of the correct prior is not obvious as the trivial allele frequency based prior was empirically found to lead to strong overcalling of the major alleles when genotype observations are missing. It is likely that the uniform prior acts as a counter balance for overcounting the alleles in the case where more than two individuals share a single allele IBD, a known problem of pairwise methods in pedigree analysis.

### IMPLEMENTATION

We implemented the SLRP program to find the approximate Maximum-A-Posteriori (MAP) estimates of the hidden variables in the Bayesian network of Figure 1, that is the phased diplotypes *h* and the IBD assignments *p*. While in principle it is possible to find the exact MAP values either deterministically, with the Junction Tree algorithm, or stochastically with Monte Carlo methods, we see that the deterministic Min-Sum algorithm provides sufficiently accurate approximations quickly and reliably.

The Min-Sum algorithm is a variant of a general class of message passing algorithms similar to e.g. Cluster Variation Method, LDPC and Turbo Code decoding, Forward-Backward, Viterbi and Loopy Belief Propagation algorithms [Durbin et al., 1998; Gallager, 1962; Kikuchi, 1951; Kschischang et al., 2001; Pearl, 1988]. The algorithm works by sending vector-valued messages along the edges of the graphical model until convergence. Intuitively, the message along an edge describes the source node's belief about the MAP value of the target node. After the message updates have converged, the incoming messages for each value *x* at each node *j* approximate the max-marginal value *M_j_*(*x*) = max
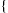
P(*y*_1_,*y*_2_,…)|*y*_*j*_ = *x*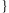. We therefore find the MAP setting by selecting the value at each node that maximize *M*_*j*_(*x*). The general message update rules along with the specific formulas for all the messages in the SLRP model are given in the supplementary material.

The messages must be updated on every step of the algorithm for all sites and pairs of individuals. This is extremely demanding even for a modest number of individuals and sites. To decrease the computational demand, we do one initial scan over all pairs of individuals to find the segments that can plausibly harbor IBD haplotypes. This initial scan is essentially a Forward–Backward [Kschischang et al., 2001] pass over each pair after which sites with more than a 50% probability of being IBD are called plausible IBD segments. The full-message passing is executed only over the plausible IBD segments which decreases the computational and memory requirements to a small fraction at the cost of not being able to call phase on sites without a recent ancestor common with someone else in the data set.

One potential issue with the Min-Sum algorithm is ensuring convergence. With appropriately dampened message updates [Murphy et al., 1999], we have not experienced these problems. In our experience, with an expected non-IBD segment length of 1 cM and an expected IBD segment length of 10 cM, the SLRP message updates converge within our default of 30 iterations.

### SIMULATED TEST DATA

To test the accuracy of SLRP, we simulated genotype data with a model resembling isolated human populations genotyped with current commercial genotyping arrays. The simulation used SimuPOP [Peng and Amos, 2008] to follow a panmictic population founded by 100 individuals growing exponentially to 18,000 in 12 generations. Recombinations were added during each mating according to the published genetic map [Myers et al., 2005] and the alleles were transmitted perfectly without mutation. We assume lack of mutations because the expected number of mutations on the observed sites during the time of the simulation is negligible (less than one observed mutation during the simulated time on all genotypes in the sample).

The simulation followed the genealogy of 7,505 SNP markers spanning the whole of chromosome 20 of length 1.09 M or 62 Mbp. The mean SNP density is thus 6,885 per Morgan. The tracked markers were chosen to be those present both in HapMap [Frazer et al., 2007] and on Illumina 370k genotyping arrays. The founding genotypes were taken from HapMap using 100 unrelated Utah residents with Northern and Western European ancestry. For each of 10 independent simulation runs, we sample a set of 190 individuals and store the identity of the founding ancestors for each tracked marker. From this information, we produce the genotypes, the true phase, and the true IBD relationships needed for the accuracy estimates.

### REAL TEST DATA

We estimate the accuracy of SLRP on real data by phasing a set of individuals recruited and genotyped as a part of the Orkney Complex Disease Study (ORCADES). ORCADES is an ongoing family-based study in the isolated Scottish archipelago of Orkney. Orkney comprises 17 inhabited islands with “Mainland” being the largest with 15,000 current inhabitants out of a total of 19,900. The genetic history of Orkney is characterized by a fairly stable population size for the last 1,000 years and by strong endogamy within different islands and villages. Most of the present day chromosomes are thought to have come to Orkney either with the Norse Viking conquest after AD 800 or via Scottish immigration in the centuries around the transfer of Orkney to Scottish rule in AD 1468. The participants recruited to the ORCADES study have a minimum of two grandparents of Orcadian ancestry, with ∼93% having three or four Orcadian grandparents [McQuillan et al., 2008; Wilson et al., 2001]. The samples were geneotyped for on Illumina HumanHap300v2 array with 310,844 markers passing QC. The average SNP density is 8,817 SNPs per Morgan.

The data set used for the evaluation consists of 599 individuals, out of whom 169 have at least one genotyped parent. We used the genotyped parents to phase the offspring on sites where the offspring was heterozygous and at least one of the parents was homozygous. None of the parents was included in the 599 individuals used for phasing so as to avoid the trivial case where SLRP is expected to excel.

We evaluate the phasing accuracy of the method on simulated and real genotyping data by comparison to Beagle [Browning and Browning, 2007] and Mach [Li et al., 2010]. We also compare accuracy of IBD detection to Beagle IBD and fastIBD and GERMLINE [Gusev et al., 2009].

## RESULTS

The described model results in significantly better phasing in isolate populations than the state of the art general purpose phasing algorithms. Simultaneously SLRP provides both the phased haplotypes and accurate IBD relationships between the phased chromosomal fragments. When comparing the different programs, one should remember that the other methods have been designed for general outbred populations and usually make a phasing and imputation call on every site, unlike SLRP which refuses to make a call when faced with too much uncertainty. If a phase call is required on every site, the SLRP output can be post processed with a general purpose phasing tool, such as IMPUTE2 [Howie et al., 2009], which can take partly phased haplotypes as input.

### PHASING ACCURACY

Table I shows the mean switch error rates and phasing yield on the simulated data for SLRP, Beagle and Mach. For complete, error free genotypes SLRP produces a full order of magnitude fewer switch errors than Beagle. Approximately 10–30% of the switch errors occur in locations where SLRP is uncertain about the phase and has left the marker unphased.

**I:** Mean number of switch errors per Morgan on simulated chromosome 20 data at sites where SLRP calls a phase

	Perfect	1% Missing	5% Missing	0.2% Errors	2% Errors
Beagle 3.0.4	63.5	68.3	98.5	73.1	182.0
Mach1	11.9	11.8	12.4	13.3	29.0
SLRP	2.7	2.8	3.4	5.1	28.8
SLRP yield (%)	92.8	92.7	91.9	87.8	45.3
SLRP within phased segments	1.7	1.7	2.1	3.6	21.0

SLRP, systematic long-range phasing.

SLRP retains most of its accuracy also when faced with noisy or missing data. Even with up to 5% missing genotypes or 0.2% genotyping error rate, SLRP results in several fold fewer switch errors than the general purpose methods. Only when the error rate becomes exceedingly high at 2% does SLRP result in a high switch error rate and low phasing yield. This feature remains also when most of the errors are on a subset of markers (20% with 10% error rate, 80% with 0.1% rate). As with other phasing and imputation methods, stringent QC of the input data is vital before applying SLRP.

Mach performs well on phasing the isolate sample, especially when faced with missing or erroneous input. While the switch error rate is typically two to three times as much as with SLRP, it is still moderate and stable over the realistic span of data missingness and error. This performance is likely due to the limited variability in our simulated sample, which can be wholly captured with the Mach model.

The SLRP model disregards the event when an individual is Homozygous By Descent (HBD), which is individuals whose two chromosomes are IBD to each other. This could result in loss of power in IBD detection and in phasing of individuals IBD to the HBD segment. We studied this by calculating the switch error rate and phasing yield on sites IBD to HBD regions. To estimate the significance of the result, we permuted the individuals 1,000 times for each independent simulation and calculated the yield and phasing error on the used sites. We observed that HBD does not have effect on phasing accuracy (*P* = 0.24) and the phasing yield is slightly better on IBD to HBD regions (93% vs. 96%, *P* = 0.04). It seems likely that the certainty of phase on homozygous regions overcomes the potential power loss due to modeling inaccuracy of HBD.

Running SLRP on an outbred population with low levels of long-range IBD does not produce useful results. When we phased 5,484 individuals from outbred European populations (WTCCC NBS and 58BC sets and HapMap CEU children using 7,293 SNP markers on chromosome 20. Density of 6,748 SNPs per Morgan), the phase was called for only 15% of the heterozygous sites.

### IBD ACCURACY

The core of the long-range phasing methods is the identification of IBD stretches from the dense genotyping data. As a by-product of phasing, SLRP outputs the inferred IBD relationships between the phased haplotypes. This information can be used further for mapping disease loci with population-based linkage analysis or IBD-mapping methods [George et al., 2000; Purcell et al., 2007].

We compare our method to three other recently published IBD detection methods, GERMLINE, Beagle IBD, and Beagle fastIBD [Browning and Browning, 2011, 2010; Gusev et al., 2009]. All of them define IBD similarly to us, as a segment of chromosome inherited in one piece from a common ancestor without internal recombinations. We use the simulated chromosome 20 data to estimate the sensitivity and the false discovery rate (FDR) for each of the methods. The results of the comparison are presented in Table II.

**II:** Median false discovery rate and sensitivity for detecting IBD on simulated chromosome 20 data

		Perfect (%)	1% Missing (%)	5% Missing (%)	0.2% Errors (%)	2% Errors (%)
Beagle	FDR	1.0	1.1	1.3	1.1	1.5
Beagle fastIBD	FDR	8.4	8.4	8.7	8.3	6.7
Germline	FDR	10.0	9.9	9.5	9.5	6.0
SLRP	FDR	1.5	1.5	1.5	1.3	1.7
Beagle	Sensitivity	12.1	13.2	22.0	19.3	13.4
Beagle fastIBD	Sensitivity	87.1	87.0	87.0	85.8	71.6
Germline	Sensitivity	69.8	68.7	62.6	65.4	10.1
SLRP	Sensitivity	68.5	68.3	67.6	55.0	17.6

SLRP, systematic long-range phasing; FDR, false-discovery rate; IBD, identity-by-descent.

The two notable features of the comparison are the high accuracy of SLRP compared to greedy pairwise methods GERMLINE and fastIBD and high sensitivity compared to Beagle IBD, which integrates over phase uncertainty. What is somewhat surprising is the poor performance of Beagle IBD on this data set. Our view is that this is because Beagle estimates background LD and haplotype frequencies from the same data that are used for IBD detection. This background estimation works when there is a negligible amount of true IBD in the data but falls apart in cases like ours, where most individuals have at least one, often many, other individuals IBD on most of the markers. In this situation, the background LD and haplotype frequencies are overestimated and accordingly the IBD probabilities are underestimated. This discrepancy highlights the difference between isolated populations and the outbred populations for which Beagle was designed.

Beagle fastIBD and to some extent GERMLINE provide good sensitivity but with a cost of FDR. The FDR advantage of SLRP is probably due to the additional power gained by requiring consistency between the haplotype phase and the IBD relationships.

### IMPUTATION ACCURACY

Most modern phasing methods are able to impute genotypes missing at random from the input data. We compared the imputation accuracy of our method to Beagle and Mach on simulated data with some genotypes set as missing. We set either 1% or 5% of the input genotypes to missing at random and ran the phasing/imputation as previously. SLRP only imputes haplotypes for which it has found a long shared segment with another individual; hence we can often only impute one allele for a missing genotype. Table III provides the error rates for the three phasing tools. The errors are counted as discordances with the observed genotype at sites that SLRP imputes fully.

**III:** Output genotype error rate on simulated chromosome 20 data at sites where SLRP imputes

	1% Missing (%)	5% Missing (%)
Beagle 3.0.4	4.04	5.34
Mach1	1.65	1.85
SLRP	0.09	0.11
Yield for alleles	76	74

SLRP, systematic long-range phasing.

SLRP excels in imputation accuracy over Beagle and Mach. While SLRP refuses to make an imputation call on about a quarter of the missing alleles, the error rate of the calls is an order of magnitude lower than with Mach, which in turn has error rate one third of that of Beagle. This accuracy is as expected given the high-phasing accuracy of SLRP as demonstrated above.

### PHASING REAL DATA

With the real data, the performance of the different methods, shown in Table IV, was similar to the results on the simulated data. SLRP performed the best by a large margin, with 3.6 switch errors on average per Morgan, followed by Mach with 17 errors per Morgan and Beagle with 23 errors per Morgan. SLRP called phase for 92% of the heterozygous sites. None of the methods showed excessive error rate biases on any chromosome.

**IV:** Switch errors per Morgan on the ORCADES data over all chromosomes

	Full data	Distantly related
Beagle 3.0.4	23.5	62.5
Mach1	17.2	23.3
SLRP	3.6	3.8
SLRP yield (%)	92	74

SLRP, systematic long-range phasing; ORCADES, Orkney Complex Disease Study.

Because of the family-based sampling in the ORCADES study, the phased set includes fairly closely related individuals even after excluding the parents. To test how the methods behave with fewer more distantly related samples, we rephased a subset of 327 individuals such that no two individuals share more than 20% of their genome as estimated by plink [Purcell et al., 2007]. This limit is equal to the expected IBD sharing of first cousins. The selected subset still includes 102 individuals with genotyped parents enabling accuracy estimation.

All the phasing methods perform worser with fewer more distantly related samples than with the closely related data set. Somewhat surprisingly, the phasing accuracy of SLRP did not drop significantly but instead the performance loss manifested as a drop in yield. While SLRP could phase 92% of the heterozygous sites with the full data, it would only phase 74% in the more distantly related data set. Of the other phasing algorithms, Mach makes a third more switch errors on the distantly related set of individuals but Beagle seems to suffer by more than doubling its error rate. While removing the closely related individuals makes the sample set conform more to the models used by Mach and Beagle, their increased error rate is likely due to decreased statistical power provided by the smaller input.

In SLRP, the expected length of the non-IBD IBS segment allows trading of the phasing accuracy for phasing yield. We tried to improve the yield by halving this value to 0.5 cM, which did improve the yield on the distantly related data set to 81% with the cost of one more switch error per Morgan. The relatively low yield in the distantly related data set as compared to our simulation is consolidated by observing that Orkney has had a stable population size for an extended period of time, resulting in well-mixed recombinant haplotypes. This is in contrast with our simulation, which has a rapidly expanding population resulting in long shared haplotype segments. It is also possible that the ORCADES data set contains an unappreciated amount of migrant haplotypes.

### COMPUTATIONAL COMPLEXITY

The complexity of the SLRP model scales linearly in the number of markers and quadratically in the number of individuals. The quadratic scaling causes the program to be quite slow on inputs with large numbers of individuals. For phasing and IBD detection in the set of 190 simulated test individuals used here, SLRP is the second fastest of the four programs after Beagle FastIBD. SLRP used 65 CPU minutes for IBD detection and phasing on average while Beagle fastIBD used 18 CPU minutes and Beagle IBD used 11 CPU minutes for phasing and 87 CPU minutes for IBD detection. The most time consuming program was Mach, which took 17 CPU hours to phase each test set. For phasing the larger ORCADES set with 599 individuals, Beagle the fastest, using one CPU hour to phase chromosome 20, followed by SLRP using 7.5 CPU hours and Mach taking 37 CPU hours.

The time and memory requirements of SLRP can be traded with the yield and accuracy by altering the model parameters, most importantly the expected non-IBD IBS segment length. Depending on the amount of IBD in the data set, SLRP spends much of the total time in message passing within the plausible IBD segments. With longer expected non-IBD segment length, the initial scan finds fewer plausible IBD segments hence there is less work to be done in the actual phasing part of the process.

Several technical tricks, such as limiting the depth of coverage of the plausible IBD segments and careful windowing of the message passing, can be used to improve the efficiency of the SLRP algorithm (see supplementary methods). Together these options have enabled us to process a large Finnish data set of more than 13,000 individuals genotyped on more than 300,000 SNP markers in 2,700 CPU hours using at most 67GB of memory.

## DISCUSSION

Inspired by the long-range phasing approach of Kong et al. [2008], we have developed a new genotype phasing model that explicitly considers IBD segments, and have shown that our implementation of the model, SLRP, gives excellent performance on simulated and real data for isolated founder populations.

It is interesting to compare the underlying model used by SLRP to that used by haplotype copying methods, such as Mach, PHASE, and IMPUTE [Howie et al., 2009]. SLRP has a quadratic number of loading variables 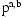 each with a small fixed size state space and number of connections, whereas Mach etc. have a linear number of loading variables, each of which can be connected to any other haplotype. The overall time complexity is similar, but SLRP explicitly models several individuals sharing a haplotype, whereas the copying methods model, at any one place, each haplotype being related to just one other.

The SLRP dependency structure is distantly inspired by the Low Density Parity Checking codes (Gallager codes) from coding theory [Gallager, 1962; Kschischang et al., 2001]. In Figure 1, the diplotypes can be seen as the received message and the IBD indicators as the parity constraints, resulting in a MAP decoding algorithm similar to our Min-Sum algorithm.

In the Bayesian network of Figure 1, it might be argued that the dependencies between the diplotypes *h* and the IBD indicators *p* should be reversed such that the diplotypes would depend on IBD status and not vice versa. While the reversal would make intuitive sense, with *n* individuals it would result in each diplotype *h* having a massive conditional probability table (CPT) with 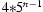 entries. In the current formulation the CPT for each *h* has only four entries. Since the size of the CPT for the IBD indicators would differ only by a constant factor (16 in fact), the formulation in Figure 1 is much more compact and hence more time and memory efficient than the alternative.

We have shown that the SLRP software and the associated probability model results in more than twofold improvement in switch error rate over general purpose phasing algorithms on samples from isolated founder populations. The IBD relations between the chromosomes are an explicit and integral part of the model and it might be possible to use those in disease gene mapping in isolate populations [George et al., 2000; Purcell et al., 2007].

### WEB RESOURCES

Software implementation of Systematic Long Range Phasing https://github.com/kpalin/SLRP
