# Supplementary material for: Identity-by-Descent-Based Phasing and Imputation in Founder Populations Using Graphical Models
Source: Genet Epidemiol. 2011 Oct 17;35(8):853–60. doi: 10.1002/gepi.20635 (PMC3368215; doi:10.1002/gepi.20635)
Supplement: Supplementary file 1 [file gepi0035-0853-SD1.pdf]

Identity by descent based phasing and imputation using graphical models Supplementary Methods

Kimmo Palin, Harry Campbell, Alan F Wright,
James F Wilson, Richard Durbin

October 14, 2011

Contents

1 SLRP formulas **2**

2 Min–Sum algorithm for MAP estimation **4**

2.1 Message equations for SLRP 6

2.2 Computational performance 7

3 Other supplemental methods **8**

1 SLRP formulas

We aim to estimate the Maximum A Posteriori configuration in the Bayesian network in Figure 1. The network has observed variables $g_j^a \in \{Hom\ 0, Het, Hom\ 1, Missing\}$ for the genotypes and hidden variables $h_j^a \in \{00, 01, 10, 11\}$ for the diplotypes and $p_j^{a,b}$ for IBD relationships. The IBD indicator variables $p_j^{a,b}$ can take values (1) IBD on first haplotypes, (2) IBD on first and second haplotype of a and b respectively, (3) IBD on second and first haplotype, (4) IBD on second haplotypes or (5) not IBD.

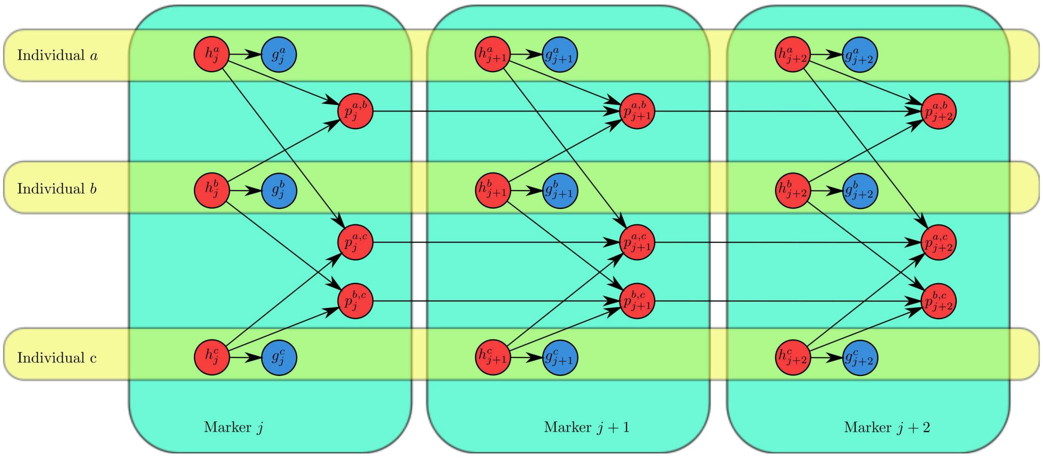

Figure 1: Bayesian network model for SLRP. Red variables are hidden, blue ones are observed.

The Bayesian network defines a probability distribution

$$\mathrm{P}(\overline{h}, \overline{p} | \overline{g}) \propto \mathrm{P}(\overline{g} | \overline{h}, \overline{p}) \mathrm{P}(\overline{h}, \overline{p}) = \mathrm{P}(\overline{g} | \overline{h}) \mathrm{P}(\overline{p} | \overline{h}) \mathrm{P}(\overline{h}) \tag{1}$$

$$= \prod_{a=0}^{n-1} \prod_{j=0}^{m-1} \mathrm{P}(g_j^a | h_j^a) \mathrm{P}(h_j^a) \prod_{b=0}^{a-1} \mathrm{P}(p_j^{a,b} | p_{j-1}^{a,b}, h_j^a, h_j^b) \tag{2}$$

Equation (2) is the factorization defined by the Bayesian network in Figure 1. The only factor that is non trivial to compute is the last one, for the probability of an IBD state given the IBD state on previous marker and the

diplotypes on current marker. This can be computed by observing that

$$\mathrm{P}(p_j^{a,b}|p_{j-1}^{a,b},h_j^a,h_j^b) = \frac{\mathrm{P}(p_j^{a,b},h_j^a,h_j^b|p_{j-1}^{a,b})}{\mathrm{P}(h_j^a,h_j^b|p_{j-1}^{a,b})} \tag{3}$$

$$= \frac{\mathrm{P}(p_j^{a,b}|p_{j-1}^{a,b})\mathrm{P}(h_j^a,h_j^b|p_j^{a,b},p_{j-1}^{a,b})}{\mathrm{P}(h_j^a,h_j^b|p_{j-1}^{a,b})} \tag{4}$$

$$= \frac{\mathrm{P}(p_j^{a,b}|p_{j-1}^{a,b})\mathrm{P}(h_j^a,h_j^b|p_j^{a,b})}{\sum_q \mathrm{P}(P_j^{a,b}=q,h_j^a,h_j^b|p_{j-1}^{a,b})} \tag{5}$$

$$= \frac{\mathrm{P}(p_j^{a,b}|p_{j-1}^{a,b})\mathrm{P}(h_j^a,h_j^b|p_j^{a,b})}{\sum_q \mathrm{P}(P_j^{a,b}=q|p_{j-1}^{a,b})\mathrm{P}(h_j^a,h_j^b|P_j^{a,b}=q)}. \tag{6}$$

Equation (3) follows from the formula for conditional probability and Equation (4) from the chain rule. Equation (5) uses the Markov property of (p_j) in the numerator and marginalisation in the denominator. Finally Equation (6) follows from application of the chain rule and Markov property in a similar manner as in equations (4) and (5).

The prior probability of diplotype $\mathrm{P}(h_j^i)$ is mostly irrelevant when the genotypes are observed since it is strongly dominated by the likelihood of the observed genotype. In the implementation the diplotype prior is essentially uniform, but with a small random noise added to break symmetries and to help convergence of the message passing algorithm. Since the noise level is low, this does not have a strong effect on the posterior but does provide an easy way to break symmetries in the model.

The "transition" probabilities $\mathrm{P}(p_j^{a,b}|p_{j-1}^{a,b})$ are given by matrix $\exp(dQ)$ where d is the genetic distance between markers $j-1$ and j and Q is the rate matrix with rate g of gaining an IBD relationship and rate l of losing IBD. This results in a rate matrix

$$Q = \begin{pmatrix} -l & 0 & 0 & 0 & l \\ 0 & -l & 0 & 0 & l \\ 0 & 0 & -l & 0 & l \\ 0 & 0 & 0 & -l & l \\ g & g & g & g & -4g \end{pmatrix} \tag{7}$$

The expected length of a non-IBD IBS segment (the expected waiting time

in the 5th state) is $(4g)^{-1}$ and the expected length of an IBD segment is l^{-1}. The probability P(IBD at site j|no IBD at state $j-1$) is additionally bound from above with the kinship coefficient given as an input parameter (default 0.04).

Conditional joint probability of diplotypes $\mathrm{P}(h_j^a, h_j^b|p)$ is the product of the allele frequencies of the freely variable alleles (three frequencies in IBD states, four in the non-IBD state) when the IBD state is consistent with the diplotypes and zero when the IBD state is inconsitent with the diplotypes. The allele frequencies are estimated from the data using the Beta–Binomial model with prior frequency distribution $Beta(1,1)$ which is the equivalent of one heterozygous "pseudoindividual".

2 Min–Sum algorithm for MAP estimation

The called phases and IBD relations are based on the *maximum posterior probability* (MAP) setting of the multidimensional distribution $\mathrm{P}(\overline{x})$, where $\overline{x} = (x_1, \ldots, x_L) = (\overline{h}, \overline{p})$ are the diplotype and IBD state variables.

We find approximate MAP settings of the variables in Figure 1 by the Min–Sum algorithm [Kschischang et al., 2001] which is a variant of a general class of message passing algorithms similar to e.g. Cluster Variation Method[Kikuchi, 1951], LDPC[Gallager, 1962] and Turbo Code[Berrou et al., 1993] decoding, Forward-Backward, Viterbi[Durbin et al., 1998] and Loopy Belief Propagation[Pearl, 1988] algorithms. Instead of maximizing the product in the right hand side of Equation (2) the algorithm minimizes the negative logarithm counterpart of it

$$\sum_{a=0}^{n-1} \sum_{j=0}^{m-1} \left(-\log \mathrm{P}(g_j^a|h_j^a) - \log \mathrm{P}(h_j^a) - \sum_{b=0}^{a-1} \log \mathrm{P}(p_j^{a,b}|p_{j-1}^{a,b}, h_j^a, h_j^b) \right) \tag{8}$$

The algorithm proceeds by iteratively updating "messages" sent along the edges between the variables (nodes) of the network in Figure 1. The algorithm is initialized by setting all messages to zero.

To describe the Min–Sum algorithm for Bayesian networks, we follow the presentation in [Kschischang et al., 2001] with notation transferred from

the $(\sum, \prod)$ semiring to $(\min, \sum)$. In Bayesian networks the messages are always a function of the parent variable. Let $\pi_Y(x)$ denote a message sent from parent X to child Y (down an edge) and $\lambda_Y(x)$ a message sent from child Y to parent X (up an edge). Intuitively the messages code for "beliefs" of the source variable about the values of the target variable. We denote the parents of a variable X by $\mathbf{A}(X)$, its children by $\mathbf{D}(X)$ and its value by x. For every variable X we have message $\lambda_X(a)$ to its parent variable $A \in \mathbf{A}(X)$, with other parents being B, C, etc.

$$\lambda_X(a) = \min_{x,b,c,\ldots} \Bigg\{ -\log \mathrm{P}\left(X = x | \mathbf{A}(X) = (a, b, \ldots)\right) + \sum_{D \in \mathbf{D}(X)} \lambda_D(x) + \pi_X(b) + \pi_X(c) + \ldots \Bigg\} \tag{9}$$

and message $\pi_D(x)$ to its child variable $D \in \mathbf{D}(X)$

$$\pi_D(x) = \min_{a,b,c,\ldots} \Bigg\{ -\log \mathrm{P}\left(X = x | \mathbf{A}(X) = (a, b, \ldots)\right) + \sum_{A \in \mathbf{A}(X)} \pi_X(a) \Bigg\} + \sum_{D \in \mathbf{D}(X) \setminus \{D\}} \lambda_D(x). \tag{10}$$

The messages are further shifted so that the minimum value of each message vector equals zero, i.e. $\min_x \pi_D(x) = \min_x \lambda_A(x) = 0$ for all D and A.

The final "beliefs" are obtained by calculating

$$BEL(x) = \min_{a,b,c,\ldots} \Bigg\{ -\log \mathrm{P}\left(X = x | \mathbf{A}(X) = (a, b, \ldots)\right) + \sum_{A \in \mathbf{A}(X)} \pi_X(a) \Bigg\} + \sum_{C \in \mathbf{D}(X)} \lambda_C(x). \tag{11}$$

whose min approximates the $-$*log maximum posterior probability* and arg min is the most probable value for x. For proofs and motivation for the formulas, see [Kschischang et al., 2001].

One potential issue with the Min-Sum algorithm is ensuring convergence. To improve it we do not use the messages calculated in Equations (9)-(10)

as is, but instead dampen the update by setting the new message to be a weighted average of the previous and the newly calculated message. The weight, or damping factor, is given by the user (default 0.75) with higher values resulting in slower but more robust convergence [Murphy et al., 1999]. With these parameters, and the default expected non-IBD segment length of 1 cM and expected IBD segment length of 10 cM, we have observed the message updates to converge within our default of 30 iterations.

2.1 Message equations for SLRP

Derived from equations (9) and (10) the Min–Sum message update formulas for the Bayesian network in Figure 1 are

$$\lambda_{G_j^a}(h_j^a) \leftarrow -\log \mathrm{P}\left(G_j^a = g_j^a | H_j^a = h_j^a\right) \tag{12}$$

$$\pi_{P_j^{a,b}}(h_j^a) \leftarrow -\log \mathrm{P}(H_j^a = h_j^a) + \lambda_{G_j^a}(h_j^a) + \sum_{x \neq b} \lambda_{P_j^{a,x}}(h_j^a) \tag{13}$$

$$\begin{aligned} \pi_{P_{j+1}^{a,b}}(p_j^{a,b}) \leftarrow \min_{p,h,h'} \Big\{ & -\log \mathrm{P}(P_j^{a,b} = p_j^{a,b} | P_{j-1}^{a,b} = p, H_j^a = h, H_j^b = h') \\ & + \pi_{P_j^{a,b}}(H_j^a = h) + \pi_{P_j^{a,b}}(H_j^b = h') + \pi_{P_j^{a,b}}(P_{j-1}^{a,b} = p) \Big\} \end{aligned} \tag{14}$$

$$\begin{aligned} \lambda_{P_j^{a,b}}(p_{j-1}^{a,b}) \leftarrow \min_{p,h,h'} \Big\{ & -\log \mathrm{P}(P_j^{a,b} = p | P_{j-1}^{a,b} = p_{j-1}^{a,b}, H_j^a = h, H_j^b = h') \\ & + \pi_{P_j^{a,b}}(H_j^a = h) + \pi_{P_j^{a,b}}(H_j^b = h') + \lambda_{P_{j+1}^{a,b}}(P_j^{a,b} = p) \Big\} \end{aligned} \tag{15}$$

$$\begin{aligned} \lambda_{P_j^{a,b}}(h_j^a) \leftarrow \min_{p,p',h} \Big\{ & -\log \mathrm{P}(P_j^{a,b} = p | P_{j-1}^{a,b} = p', H_j^a = h_j^a, H_j^b = h) \\ & + \pi_{P_j^{a,b}}(P_{j-1}^{a,b} = p') + \pi_{P_j^{a,b}}(H_j^b = h) + \lambda_{P_{j+1}^{a,b}}(P_j^{a,b} = p) \Big\} \end{aligned} \tag{16}$$

The messages in Equations (12) – (16) are updated a predefined (default 30) number of times. New message values do not completely replace the old ones but the updates are "dampened"by calculating a weighted average of new and old messages, with a predefined damping factor of 0.75 contribution of old messages.[Murphy et al., 1999]

The MAP phase for a diplotype H_j^a is found as

$$\arg\min \left\{ -\log \mathrm{P}(H_j^a = h_j^a) + \lambda_{G_j^a}(h_j^a) + \sum_x \lambda_{P_j^{a,x}}(h_j^a) \right\}$$

which approximately corresponds to the max–marginal of the diplotype, i.e. most probable phase when other variables are set to their maximal value.

A diplotype phase is not called if the two most probable phases are within a defined MAP probability ratio of each other (default 2). The implementation stores only the current diplotype beliefs and the messages coming from the IBD variables to the diplotype variables.

The Min–Sum algorithm is executed only over markers and pairs of individuals that are found IBD during preprocessing.

2.2 Computational performance

To enable phasing of large datasets including tens of thousands of individuals, the implementation includes features to distribute the workload over multiple processors on shared and non-shared memory architectures using threads or MPI. Furthermore there are options to decrease the memory requirement by using single, instead of double, precision floating point numbers and by doing the message passing in a wave over the chromosome. Also, there is an option to to prune the set of plausible IBD segments such that each site is covered at least by given number of most likely plausible IBD segments. Briefly, in the pruning mode, we search, for each individual, all plausible IBD segments and retain the most likely such that each site is covered by the given number of segments, if possible. In essence, this method provides a soft lower bound for depth of coverage of plausible IBD segments. In practice, most sites seem to be covered by 2-3 times the limit coverage, when given sufficiently large number of individuals to phase. Neither the wave or the pruning modes were used in the experiments presented in the main paper.

In the wave mode, illustrated in Figure 2, the message passing is executed only on the plausible IBD segments overlapping a fixed width segment of the chromosome (the wave) up to the leading edge but including parts prior to

the trailing edge of the wave. This way the software needs to store only a limited number of messages without causing severe discontinuities on the segment boundaries.

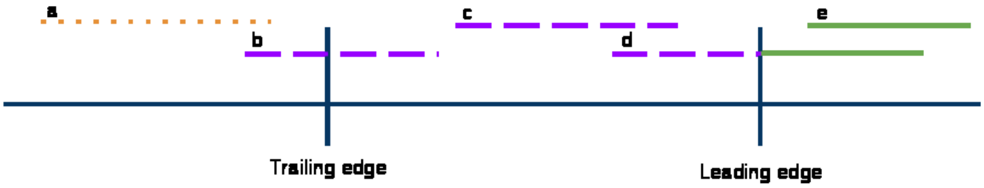

Figure 2: Wave mode for message passing.
Plausible IBD segments $a-e$ for one pair of individuals. The wave is between the trailing and the leading edge. The messages are passed on the dashed magenta segments b, c and d. The dotted segment a has already been processed. Solid green segments e and part of d are not yet being processed.

3 Other supplemental methods

For ORCADES, pedigree, surname and genetic analyses [McQuillan et al., 2008, Wilson et al., 2001] indicate that a very high proportion of their ancestry dates back centuries in Orkney; the descendants of late 20th century immigrants are excluded from the study. Genetic diversity in this population is decreased compared to Mainland Scotland, consistent with the historically high levels of endogamy. Data for participants aged 18-100 years, from a subgroup of ten islands, were used for this analysis. All participants gave informed consent and the study was approved by Research Ethics Committees in Orkney and Aberdeen.

A total of 151 individuals out of the 599 in the ORCADES dataset have at least one full or half sibling in the dataset.

Mach was run according to instructions in the README file with 200 states and 50 rounds.

Beagle phasing used version 3.0.2 with 20 iterations instead of the default 10 to improve the phasing accuracy. GERMLINE was run as instructed in

the user manual on Beagle phased haplotypes with the length of the called IBD segments limited to 5cM. The missing genotypes were imputed by Beagle during phasing.

Beagle IBD detection was done with version 3.2 with the default number of iterations for the background haplotype distribution estimation. The parameters ibd2nonibd and nonibd2ibd were estimated from the simulation outcome and the true values were given to the program (`ibd2nonibd=0.187015` and `nonibd2ibd=0.00926624`). The genetic map given to Beagle was identical to the one used to generate the data.

Beagle fastIBD was run as ten times with distinct random seeds 1-10. The output fibd files were processed with process_fibd.py by Sharon Browning (http://faculty.washington.edu/sguy/beagle/ibd_and_hbd/ibd_and_hbd.html). The results for different thresholds (used in post processing) are given in Table 3. The results reported in the main text are for threshold 10^{-6} which was recomended in the fastIBD paper.

Threshold	Type	FDR(median)	Sensitivity(median)
1.00E-06		15%	93%
1.00E-06	1% Missing	15%	93%
1.00E-06	5% Missing	17%	93%
1.00E-06	0.2% Error	15%	92%
1.00E-06	2% Error	19%	86%
1.00E-10		8%	87%
1.00E-10	1% Missing	8%	87%
1.00E-10	5% Missing	9%	87%
1.00E-10	0.2% Error	8%	86%
1.00E-10	2% Error	7%	72%
1.00E-20		5%	72%
1.00E-20	1% Missing	5%	72%
1.00E-20	5% Missing	5%	71%
1.00E-20	0.2% Error	5%	69%
1.00E-20	2% Error	3%	35%
1.00E-30		4%	59%
1.00E-30	1% Missing	4%	59%
1.00E-30	5% Missing	4%	57%
1.00E-30	0.2% Error	4%	55%
1.00E-30	2% Error	2%	14%
1.00E-40		4%	48%
1.00E-40	1% Missing	4%	47%
1.00E-40	5% Missing	4%	45%
1.00E-40	0.2% Error	3%	43%
1.00E-40	2% Error	2%	6%

Table 1: False discovery rates and sensitivities for different thresholds with fastIBD method

References

[Berrou et al., 1993] Berrou, C., Glavieux, A., and Thitimajshima, P. (1993). Near Shannon limit error-correcting coding and decoding: Turbo-codes. 1. In *Proc IEEE International Conference on Communications*, volume 2, pages 1064–1070. IEEE.

[Browning and Browning, 2011] Browning, B. L. and Browning, S. R. (2011). A fast, powerful method for detecting identity by descent. *American journal of human genetics*, 88(2):173–82.

[Durbin et al., 1998] Durbin, R., Eddy, S., Krogh, A., and Mitchison, G. (1998). *Biological sequence analysis: Probabilistic models of proteins and nucleic acids.* Cambridge University Press.

[Gallager, 1962] Gallager, R. (1962). Low-density parity-check codes. *IRE Transactions on Information Theory*, 8(1):21–28.

[Kikuchi, 1951] Kikuchi, R. (1951). A Theory of Cooperative Phenomena. *Physical Review*, 81(6):988–1003.

[Kschischang et al., 2001] Kschischang, F. R., Frey, B. J., and Loeliger, H. A. (2001). Factor graphs and the Sum-Product algorithm. *IEEE TRANSACTIONS ON INFORMATION THEORY*, 47(2).

[Li and Stephens, 2003] Li, N. and Stephens, M. (2003). Modeling Linkage Disequilibrium and Identifying Recombination Hotspots Using Single-Nucleotide Polymorphism Data. *Genetics*, 165(4):2213–2233.

[McQuillan et al., 2008] McQuillan, R., Leutenegger, A.-L., Abdel-Rahman, R., Franklin, C. S., Pericic, M., Barac-Lauc, L., Smolej-Narancic, N., Janicijevic, B., Polasek, O., Tenesa, A., Macleod, A. K., Farrington, S. M., Rudan, P., Hayward, C., Vitart, V., Rudan, I., Wild, S. H., Dunlop, M. G., Wright, A. F., Campbell, H., and Wilson, J. F. (2008). Runs of homozygosity in European populations. *American journal of human genetics*, 83(3):359–72.

[Murphy et al., 1999] Murphy, K., Weiss, Y., and Jordan, M. (1999). Loopy belief propagation for approximate inference: An empirical study. In *Proceedings of Uncertainty in AI*, pages 467–475. Morgan Kaufmann.

[Pearl, 1988] Pearl, J. (1988). *Probabilistic reasoning in intelligent systems: networks of plausible inference.* Morgan Kaufmann.

[Wilson et al., 2001] Wilson, J. F., Weiss, D. A., Richards, M., Thomas, M. G., Bradman, N., and Goldstein, D. B. (2001). Genetic evidence for different male and female roles during cultural transitions in the British Isles. *Proceedings of the National Academy of Sciences of the United States of America*, 98(9):5078–83.
